# Contemporary use of oesophageal temperature probes during left atrial ablation: results from an EHRA Scientific Initiatives Committee survey

**DOI:** 10.1093/europace/euag255

**Published:** 2026-10-06

**Authors:** Arian Sultan, Piotr Futyma, Andreas Metzner, Giacomo Mugnai, Mark T Mills, Martin H Ruwald, Laura Perrotta, K R Julian Chun, the EHRA Scientific Initiatives Committee, K R Julian Chun, Laura Perrotta, Arian Sultan

**Affiliations:** Department of Electrophysiology, St. Georg Heart Centre Hamburg, Asklepios Clinic Hamburg, Lohmühlenstraße 5, Hamburg 20099, Germany; Faculty of Medicine University of Cologne, Cologne, Germany; St. Joseph’s Heart Rhythm Centre, Rzeszów, Poland; Medical College, University of Rzeszów, Rzeszów, Poland; University Heart and Vascular Centre Hamburg, University Medical Centre Hamburg-Eppendorf, Hamburg, Germany; Electrophysiology and Cardiac Pacing, University Hospital of Verona, Verona, Italy; Department of Cardiology, Sheffield Teaching Hospitals NHS Foundation Trust, Sheffield, UK; Division of Electrophysiology, Department of Cardiology, Herlev-Gentofte Hospital, Hellerup, Denmark; Azienda Ospedaliero-Universitaria Careggi, Florence, Italy; MVZ CCB Frankfurt und Main-Taunus GbR, Frankfurt, Germany; Klinik für Rhythmologie, University Hospital Schleswig-Holstein, Lübeck, Germany

**Keywords:** Atrial fibrillation, Oesophageal temperature monitoring, Atrio-oesophageal fistula, Catheter ablation, Pulsed field ablation, EHRA survey

## Introduction

Atrio-oesophageal fistula is a rare but frequently fatal complication of left atrial ablation. Its reported incidence ranges from 0.03 to 0.2%, with mortality rates of 50–80% and therefore constitutes one of the most severe complications of catheter ablation.^1–3^ Oesophageal temperature monitoring is commonly used during radiofrequency and cryoballoon ablation to detect thermal exposure and prompt interruption or modification of energy delivery. However, randomized and observational data have not established that esophageal temperature probes (ETPs) prevent oesophageal injury or atrial esophageal fistula (AEF), and results may be influenced by probe design, positioning, and temperature thresholds.^4–6^ The increasing use of pulsed field ablation (PFA) has further changed oesophageal protection strategies.^7,8^

The EHRA Scientific Initiatives Committee therefore conducted an international survey to describe contemporary ETP use during left atrial ablation.

## Methods

A 35-item online questionnaire was developed by the EHRA Scientific Initiatives Committee and distributed through EHRA channels, national cardiac and electrophysiology societies, social media, and the EHRA Scientific Research Network. The survey was available for 6 weeks from April 2024. Participation was physician-based, anonymous, and voluntary.

Questions addressed centre characteristics, procedural volumes, sedation, preprocedural imaging, energy sources, ETP type and deployment, temperature thresholds, management of temperature excursions, experience with AEF, and anticipated future ETP use. Data are presented descriptively.

## Results

A total of 232 physicians from 37 countries completed the survey, predominantly from Europe. Respondents worked in university hospitals (48%), non-university hospitals (37%), and private hospitals or practices (15%). Annual catheter ablation volume was 301–999 procedures in 43% of centres, 101–300 in 27.6%, >1000 in 16%, and <100 in ∼10%.

For *de novo* pulmonary vein isolation (PVI), 62% reported performing 50–300 procedures annually, approximately one-quarter performed 301–600, 9% performed 601–1000, and 5% performed >1000. For complex left atrial ablation beyond PVI, 66% performed 50–300 procedures annually, 22.4% performed 301–700, 5% performed 701–1000, and 6% performed >1000.

Analgo-sedation was used by 88.5% for PVI and PVI-plus procedures; one-third reported routine general anaesthesia, and 11.5% performed procedures under local anaesthesia in conscious patients. Preprocedural imaging was routinely used by 62%, most commonly transoesophageal echocardiography and computed tomography. However, 94% reported that oesophageal proximity to the posterior left atrium did not influence ETP deployment; only 6% used imaging findings to trigger ETP use. Oesophageal deviation devices were used by 12%.

The CIRCA S-Cath was the most frequently used ETP (51%), followed by FIAB probes (26%); 33% reported using other devices. ETPs were used predominantly during thermal ablation, including radiofrequency and cryoballoon procedures, and infrequently during PFA. The main reasons for non-use were lack of evidence for AEF prevention (56%), cost (31%), increasing use of PFA (29%), concern regarding an antenna effect (26%), and complications or patient intolerance (22%).

Reported upper temperature thresholds ranged from 30 to 48°C and lower thresholds from 10 to 20°C. When thresholds were exceeded, 68% modified the ablation strategy, including power reduction, shorter energy delivery, interruption of ablation, or catheter repositioning. Only 16% routinely performed endoscopy or intensified proton-pump inhibitor therapy.

Twenty-five per cent of respondents had encountered at least one AEF, most frequently after radiofrequency ablation, followed by cryoballoon and laser ablation. Regarding future use, two-thirds anticipated a minimal or absent role for ETPs; fewer than 10% expected their role to increase. A condensed summary is depicted in *Figure 1*.

**Figure 1:**
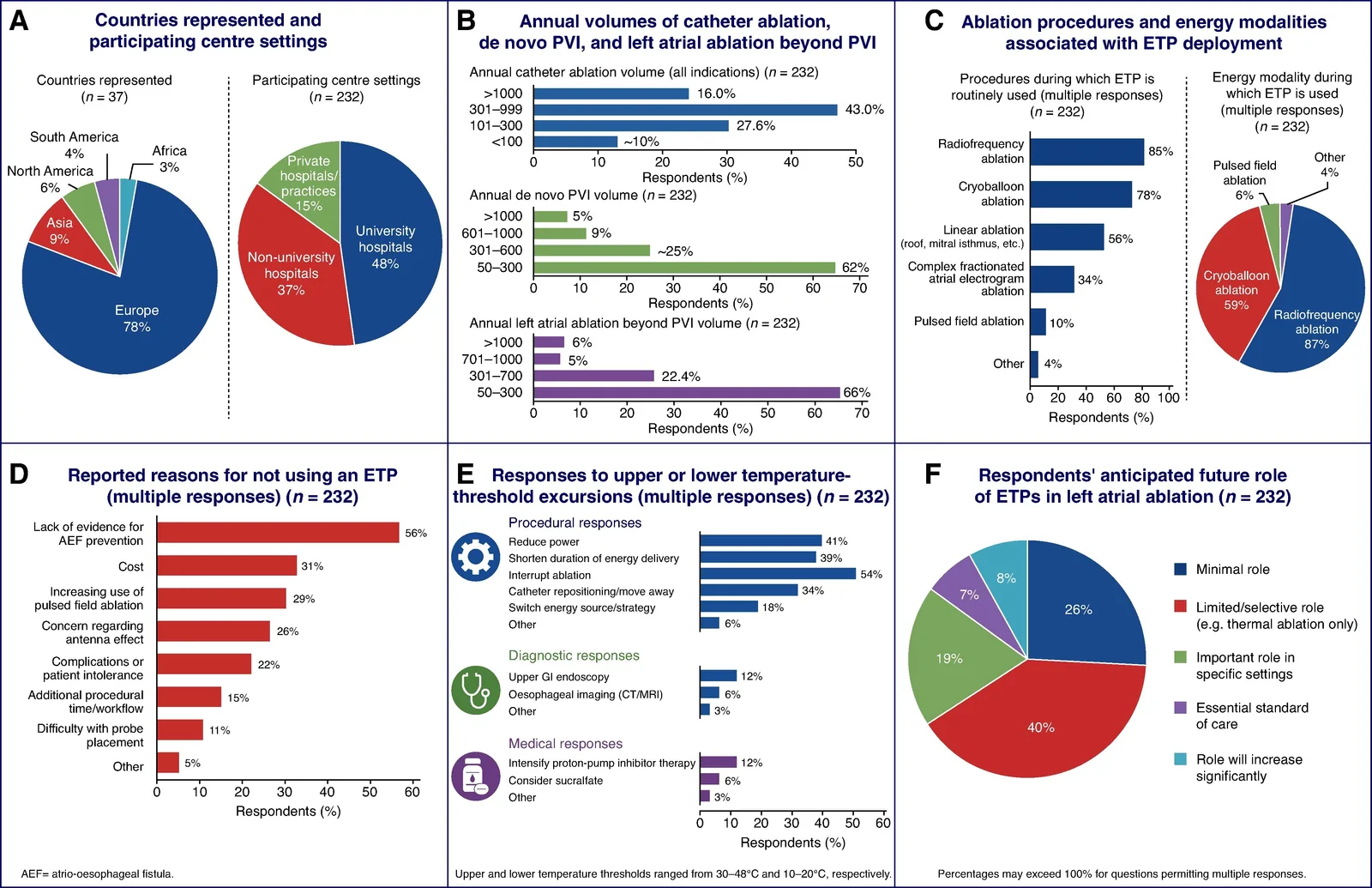
Contemporary use of oesophageal temperature probes during left atrial ablation. (*A*) Countries represented and participating centre settings. (*B*) Annual volumes of catheter ablation, *de novo* PVI, and left atrial ablation beyond PVI. (*C*) Ablation procedures and energy modalities associated with ETP deployment. (*D*) Reported reasons for not using an ETP. (*E*) Procedural, diagnostic, and medical responses to upper or lower temperature-threshold excursions. (F) Respondents’ anticipated future role of ETPs in left atrial ablation. Percentages may exceed 100% for questions permitting multiple responses.

## Discussion

This survey documents substantial variation in oesophageal temperature monitoring during left atrial ablation. Although routine preprocedural imaging was common, oesophageal proximity rarely determined ETP deployment. ETPs were used mainly with thermal energy, while PFA was a frequent reason for non-use. Temperature thresholds differed considerably between centres, and threshold excursions most often resulted in procedural modification. Additional endoscopic evaluation or intensified medical therapy was reported less frequently.

The reported practice patterns occur in the context of randomized and pooled analyses that have not demonstrated prevention of oesophageal injury with ETP monitoring.^4–6^ Previous cryoballoon studies have nevertheless described associations between luminal oesophageal temperature and endoscopically detected lesions; however, temperature assessment varies throughout different probe manufacturers.^9,10^ PFA studies have reported limited oesophageal injury compared with thermal energy sources, while changes in intraluminal oesophageal temperature during PFA have also been documented.^7,8^

Participation was voluntary and may have favoured physicians with a specific interest in oesophageal protection. Responses were predominantly European and included a large proportion from Germany. Some practices, including sedation and staffing, may be influenced by national regulations and local infrastructure. Survey responses represent reported practice rather than independently verified procedural data.

## Conclusion

In this international EHRA survey, ETP use was heterogeneous, largely restricted to thermal ablation, and infrequently guided by preprocedural imaging. Temperature excursions primarily prompted modification of ablation delivery, while additional diagnostic or medical management was uncommon.

## Data Availability

The data underlying this article are available from the corresponding author upon reasonable request.
